# 2,5-Dimethyl­hexane-2,5-diyl bis­(4-nitro­phen­yl) dicarbonate

**DOI:** 10.1107/S1600536808006120

**Published:** 2008-03-12

**Authors:** Syed Nawazish Ali, Sabira Begum, Mitchell A. Winnik, Alan J. Lough

**Affiliations:** a HEJ Research Institute of Chemistry, International Center for Chemical Sciences, University of Karachi, Karachi 75270, Pakistan; b Department of Chemistry, University of Toronto, Toronto, Ontario, Canada M5S 3H6

## Abstract

The title structure, C_22_H_24_N_2_O_10_, contains two independent centrosymmetric mol­ecules. The only significant difference between the mol­ecules is the dihedral angle between the unique carbonate group (–O—CO_2_–) and the benzene ring, the values being 77.35 (8) and 66.42 (8)°. The crystal structure is stabilized by weak inter­molecular C—H⋯O hydrogen bonds.

## Related literature

For related literature, see: Nawazish Ali *et al.* (2008 ▶).
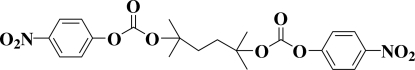


## Experimental

### 

#### Crystal data

C_22_H_24_N_2_O_10_
*M* *_r_* = 476.43
Triclinic, 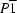

*a* = 8.1829 (4) Å
*b* = 11.7024 (5) Å
*c* = 11.8928 (6) Å
α = 92.717 (3)°
β = 98.886 (2)°
γ = 92.952 (3)°
*V* = 1121.92 (9) Å^3^
*Z* = 2
Mo *K*α radiation
μ = 0.11 mm^−1^
*T* = 150 (2) K
0.34 × 0.25 × 0.14 mm

#### Data collection

Bruker–Nonius KappaCCD diffractometer
Absorption correction: multi-scan (*SORTAV*; Blessing, 1995 ▶) *T*_min_ = 0.803, *T*_max_ = 0.985
9940 measured reflections
5069 independent reflections
3571 reflections with *I* > 2σ(*I*)
*R*_int_ = 0.047

#### Refinement

*R*[*F* ^2^ > 2σ(*F* ^2^)] = 0.050
*wR*(*F* ^2^) = 0.132
*S* = 1.04
5069 reflections
311 parameters
H-atom parameters constrained
Δρ_max_ = 0.23 e Å^−3^
Δρ_min_ = −0.28 e Å^−3^

### 

Data collection: *COLLECT* (Nonius, 2002 ▶); cell refinement: *DENZO-SMN* (Otwinowski & Minor, 1997 ▶); data reduction: *DENZO-SMN*; program(s) used to solve structure: *SIR92* (Altomare *et al.*, 1994 ▶); program(s) used to refine structure: *SHELXTL* (Sheldrick, 2008 ▶); molecular graphics: *SHELXTL*; software used to prepare material for publication: *SHELXTL*.

## Supplementary Material

Crystal structure: contains datablocks global, I. DOI: 10.1107/S1600536808006120/pv2070sup1.cif

Structure factors: contains datablocks I. DOI: 10.1107/S1600536808006120/pv2070Isup2.hkl

Additional supplementary materials:  crystallographic information; 3D view; checkCIF report

## Figures and Tables

**Table 1:** Hydrogen-bond geometry (Å, °)

*D*—H⋯*A*	*D*—H	H⋯*A*	*D*⋯*A*	*D*—H⋯*A*
C5*A*—H5*AA*⋯O5*B*^i^	0.95	2.59	3.112 (2)	115
C5*B*—H5*BA*⋯O5*A*^ii^	0.95	2.52	3.205 (2)	129
C11*A*—H11*B*⋯O3*A*^iii^	0.99	2.53	2.888 (2)	101
C11*A*—H11*B*⋯O4*B*^iii^	0.99	2.54	3.502 (2)	165
C11*B*—H11*C*⋯O4*B*^iv^	0.99	2.58	3.546 (2)	164
C11*B*—H11*D*⋯O3*B*^v^	0.99	2.49	2.862 (2)	102

Symmetry codes: (i) 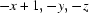; (ii) 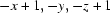; (iii) 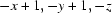; (iv) 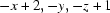; (v) 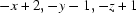.

**Fig. 1.**
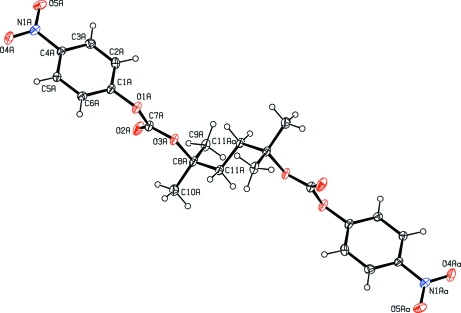
The molecular structure of molecule A showing displacement ellipsoids drawn at the 30% probability level [symmetry code: (a) -x + 1, -y + 1, -z]

**Fig. 2.**
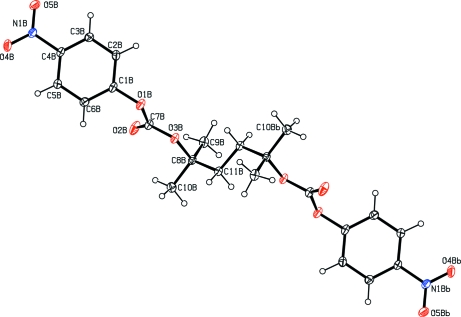
The molecular structure of molecule B showing displacement ellipsoids drawn at the 30% probability level. [symmetry code: (b) -x + 2, -y - 1, -z + 1]

**Fig. 3.**
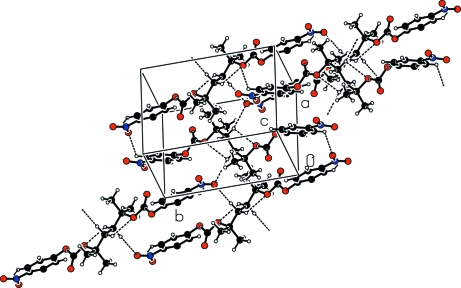
Part of the crystal structure with weak C—H···O hydrogen bonds shown as dashed lines.

## supplementary crystallographic information

### Comment

For background information and relevant references see Ali *et al.* (2008). The title structure contains two independent centrosymmetric molecules labelled with suffix A and suffix B to indicate molecules A and B (see Figs. 1 and 2). The only significant difference between them is a slight difference in the dihedral angles in each molecule, between the unique carbonate group (O1/O2/O3/C7) and benzene ring (C1—C6) which is 77.35 (8)° for molecule A and 66.42 (8)° for molecule B. In addition to weak intramolecular C—H···O hydrogen bonds which may, in part, affect the conformation of each molecule, the crystal structure is stabilized by weak intermolecular hydrogen bonds (see Fig. 3).

### Experimental

A solution of 4-nitrophenylchloroformate (2.02 g, 10.0 mmol) in dry dichloromethane (25 ml) was added dropwise *via* a 100 ml separatory funnel into a solution of 2,5-dimethyl-2,5-hexanediol (0.74 g, 5.0 mmol) in anhydrous pyridine (0.70 g, 0.72 ml, 8.8 mmol) and dry dichloromethane (15 ml) in a 100 ml round-bottom flask. A white suspension appeared which was allowed to stir gently at room temperature for 14 h. After this time more dry dichloromethane (40 ml) was added, which dissolved the suspension and then the reaction mixture was stirred for another 6 h. Then it was quenched by adding deionized water (40 ml). The reaction mixture was transferred to a separatory funnel (250 ml), and the lower organic phase was removed. The aqueous phase was washed with dichloromethane (25 ml *x* 2), and all the dichloromethane solutions were combined. These were then washed with deionized water (40 ml *x* 2), a 1.0% solution of acetic acid (40 ml *x* 3) and once more with deionized water (30 ml *x* 2), and then dried over anhydrous magnesium sulfate and filtered. After filtration, the solvent was removed by rotary evaporator. The product was dried in air overnight in a fume hood and then in a vacuum oven for 24 h at room temperature (< 1 Torr). The desired product was obtained in a good yield (29.4 g, 90.1%) as a white solid; the product was recrystallized in dichloromethane. X-ray quality crystals were obtained after the slow evaporation of a solution of the title compound at room temperature.

### Refinement

All hydrogen atoms were placed in calculated positions with C—H = 0.95 - 0.99 Å and they were included in the refinement in the riding-model approximation with *U*_iso_(H) = 1.2*U*_eq_(C) or 1.5*U*_eq_(C) for methyl C atoms.

### Crystal data

**Table:**

C_22_H_24_N_2_O_10_	*Z* = 2
*M**_r_* = 476.43	*F*_000_ = 500
Triclinic, *P*1	*D*_x_ = 1.410 Mg m^−^^3^
Hall symbol: -P 1	Mo *K*α radiation λ = 0.71073 Å
*a* = 8.1829 (4) Å	Cell parameters from 9940 reflections
*b* = 11.7024 (5) Å	θ = 2.8–27.5º
*c* = 11.8928 (6) Å	µ = 0.11 mm^−^^1^
α = 92.717 (3)º	*T* = 150 (2) K
β = 98.886 (2)º	Needle, colourless
γ = 92.952 (3)º	0.34 × 0.25 × 0.14 mm
*V* = 1121.92 (9) Å^3^	

### Data collection

**Table:**

Bruker–Nonius KappaCCD diffractometer	5069 independent reflections
Radiation source: fine-focus sealed tube	3571 reflections with *I* > 2σ(*I*)
Monochromator: graphite	*R*_int_ = 0.047
Detector resolution: 9 pixels mm^-1^	θ_max_ = 27.5º
*T* = 150(2) K	θ_min_ = 2.8º
φ scans and ω scans with κ offsets	*h* = −10→10
Absorption correction: multi-scan(SORTAV; Blessing, 1995)	*k* = −15→15
*T*_min_ = 0.803, *T*_max_ = 0.985	*l* = −15→15
9940 measured reflections	

### Refinement

**Table:**

Refinement on *F*^2^	Secondary atom site location: difference Fourier map
Least-squares matrix: full	Hydrogen site location: inferred from neighbouring sites
*R*[*F*^2^ > 2σ(*F*^2^)] = 0.050	H-atom parameters constrained
*wR*(*F*^2^) = 0.132	*w* = 1/[σ^2^(*F*_o_^2^) + (0.0508*P*)^2^ + 0.4455*P*] where *P* = (*F*_o_^2^ + 2*F*_c_^2^)/3
*S* = 1.04	(Δ/σ)_max_ = 0.001
5069 reflections	Δρ_max_ = 0.23 e Å^−^^3^
311 parameters	Δρ_min_ = −0.28 e Å^−^^3^
Primary atom site location: structure-invariant direct methods	Extinction correction: none

### Special details

**Table:**

Experimental. multi-scan from symmetry-related measurements *SORTAV* (Blessing 1995)
Geometry. All e.s.d.'s (except the e.s.d. in the dihedral angle between two l.s. planes) are estimated using the full covariance matrix. The cell e.s.d.'s are taken into account individually in the estimation of e.s.d.'s in distances, angles and torsion angles; correlations between e.s.d.'s in cell parameters are only used when they are defined by crystal symmetry. An approximate (isotropic) treatment of cell e.s.d.'s is used for estimating e.s.d.'s involving l.s. planes.
Refinement. Refinement of *F*^2^ against ALL reflections. The weighted *R*-factor *wR* and goodness of fit *S* are based on *F*^2^, conventional *R*-factors *R* are based on *F*, with *F* set to zero for negative *F*^2^. The threshold expression of *F*^2^ > σ(*F*^2^) is used only for calculating *R*-factors(gt) *etc*. and is not relevant to the choice of reflections for refinement. *R*-factors based on *F*^2^ are statistically about twice as large as those based on *F*, and *R*- factors based on ALL data will be even larger.

### Fractional atomic coordinates and isotropic or equivalent isotropic displacement parameters (Å^2^)

**Table:**

	*x*	*y*	*z*	*U*_iso_*/*U*_eq_	
O1A	0.45560 (16)	0.14403 (11)	0.12248 (12)	0.0314 (3)	
O2A	0.19552 (18)	0.20327 (12)	0.11199 (15)	0.0434 (4)	
O3A	0.40180 (16)	0.30199 (10)	0.04414 (12)	0.0287 (3)	
O4A	0.3070 (2)	−0.32743 (12)	0.32493 (14)	0.0421 (4)	
O5A	0.33546 (19)	−0.21668 (14)	0.47827 (13)	0.0415 (4)	
N1A	0.3299 (2)	−0.23269 (14)	0.37471 (15)	0.0307 (4)	
C1A	0.4142 (2)	0.05054 (15)	0.18498 (16)	0.0250 (4)	
C2A	0.3979 (3)	0.06818 (16)	0.29791 (18)	0.0310 (5)	
H2AA	0.4071	0.1437	0.3323	0.037*	
C3A	0.3682 (2)	−0.02484 (17)	0.36098 (17)	0.0299 (4)	
H3AA	0.3569	−0.0146	0.4391	0.036*	
C4A	0.3553 (2)	−0.13300 (15)	0.30750 (16)	0.0238 (4)	
C5A	0.3715 (2)	−0.15112 (16)	0.19471 (16)	0.0273 (4)	
H5AA	0.3621	−0.2265	0.1602	0.033*	
C6A	0.4016 (2)	−0.05742 (16)	0.13248 (17)	0.0277 (4)	
H6AA	0.4135	−0.0676	0.0545	0.033*	
C7A	0.3338 (2)	0.21770 (15)	0.09375 (17)	0.0279 (4)	
C8A	0.3008 (2)	0.39869 (15)	0.00122 (17)	0.0267 (4)	
C9A	0.2301 (3)	0.45775 (16)	0.09795 (18)	0.0333 (5)	
H9AA	0.1359	0.4106	0.1157	0.050*	
H9AB	0.1930	0.5327	0.0751	0.050*	
H9AC	0.3158	0.4681	0.1656	0.050*	
C10A	0.1670 (3)	0.35357 (17)	−0.09497 (19)	0.0351 (5)	
H10A	0.0844	0.3054	−0.0646	0.053*	
H10B	0.2164	0.3080	−0.1508	0.053*	
H10C	0.1133	0.4180	−0.1319	0.053*	
C11A	0.4289 (2)	0.47525 (15)	−0.04470 (16)	0.0267 (4)	
H11A	0.4752	0.4303	−0.1037	0.032*	
H11B	0.3722	0.5393	−0.0822	0.032*	
O1B	0.96232 (16)	−0.15529 (11)	0.35566 (12)	0.0303 (3)	
O2B	0.70189 (17)	−0.19261 (12)	0.39066 (14)	0.0420 (4)	
O3B	0.91508 (15)	−0.30493 (10)	0.44521 (11)	0.0261 (3)	
O4B	0.8268 (2)	0.33919 (12)	0.20273 (13)	0.0450 (4)	
O5B	0.82071 (18)	0.25161 (11)	0.03819 (12)	0.0338 (3)	
N1B	0.8330 (2)	0.25233 (13)	0.14248 (14)	0.0272 (4)	
C1B	0.9163 (2)	−0.05454 (15)	0.30242 (17)	0.0252 (4)	
C2B	0.9283 (2)	−0.05045 (15)	0.18834 (17)	0.0274 (4)	
H2BA	0.9575	−0.1159	0.1471	0.033*	
C3B	0.8973 (2)	0.05059 (15)	0.13453 (16)	0.0263 (4)	
H3BA	0.9031	0.0553	0.0557	0.032*	
C4B	0.8580 (2)	0.14393 (14)	0.19799 (16)	0.0235 (4)	
C5B	0.8458 (2)	0.14058 (16)	0.31213 (16)	0.0265 (4)	
H5BA	0.8184	0.2064	0.3535	0.032*	
C6B	0.8747 (2)	0.03886 (16)	0.36517 (17)	0.0285 (4)	
H6BA	0.8659	0.0335	0.4435	0.034*	
C7B	0.8420 (2)	−0.21728 (15)	0.39835 (17)	0.0265 (4)	
C8B	0.8149 (2)	−0.39774 (15)	0.49142 (16)	0.0238 (4)	
C9B	0.6938 (2)	−0.45765 (16)	0.39468 (18)	0.0313 (5)	
H9BA	0.6058	−0.4066	0.3693	0.047*	
H9BB	0.6450	−0.5278	0.4211	0.047*	
H9BC	0.7521	−0.4773	0.3310	0.047*	
C10B	0.7291 (3)	−0.34772 (17)	0.58539 (18)	0.0326 (5)	
H10D	0.6399	−0.3011	0.5521	0.049*	
H10E	0.8095	−0.2998	0.6396	0.049*	
H10F	0.6825	−0.4101	0.6251	0.049*	
C11B	0.9494 (2)	−0.47459 (15)	0.54251 (16)	0.0253 (4)	
H11C	1.0257	−0.4294	0.6031	0.030*	
H11D	0.8965	−0.5381	0.5787	0.030*	

### Atomic displacement parameters (Å^2^)

**Table:**

	*U*^11^	*U*^22^	*U*^33^	*U*^12^	*U*^13^	*U*^23^
O1A	0.0291 (7)	0.0220 (7)	0.0461 (9)	0.0043 (5)	0.0117 (6)	0.0132 (6)
O2A	0.0266 (8)	0.0360 (8)	0.0714 (12)	0.0030 (6)	0.0125 (8)	0.0251 (8)
O3A	0.0282 (7)	0.0183 (6)	0.0414 (8)	0.0018 (5)	0.0084 (6)	0.0096 (6)
O4A	0.0550 (10)	0.0235 (8)	0.0503 (10)	−0.0004 (7)	0.0155 (8)	0.0077 (7)
O5A	0.0457 (9)	0.0531 (10)	0.0296 (8)	0.0092 (7)	0.0114 (7)	0.0165 (7)
N1A	0.0301 (9)	0.0307 (9)	0.0337 (10)	0.0059 (7)	0.0079 (7)	0.0117 (8)
C1A	0.0237 (10)	0.0199 (9)	0.0322 (11)	0.0028 (7)	0.0047 (8)	0.0081 (8)
C2A	0.0342 (11)	0.0217 (9)	0.0377 (12)	0.0015 (8)	0.0089 (9)	−0.0037 (8)
C3A	0.0324 (11)	0.0332 (11)	0.0253 (10)	0.0048 (8)	0.0073 (8)	0.0013 (8)
C4A	0.0219 (9)	0.0231 (9)	0.0269 (10)	0.0035 (7)	0.0033 (8)	0.0072 (8)
C5A	0.0338 (11)	0.0201 (9)	0.0277 (10)	0.0021 (8)	0.0035 (8)	0.0013 (8)
C6A	0.0332 (11)	0.0268 (10)	0.0236 (10)	0.0015 (8)	0.0050 (8)	0.0043 (8)
C7A	0.0301 (11)	0.0194 (9)	0.0347 (11)	0.0008 (8)	0.0062 (9)	0.0047 (8)
C8A	0.0292 (10)	0.0183 (9)	0.0332 (11)	0.0034 (7)	0.0041 (8)	0.0078 (8)
C9A	0.0367 (12)	0.0233 (10)	0.0425 (13)	0.0021 (8)	0.0138 (10)	0.0039 (9)
C10A	0.0339 (11)	0.0282 (10)	0.0414 (13)	−0.0031 (9)	0.0008 (10)	0.0046 (9)
C11A	0.0311 (10)	0.0199 (9)	0.0295 (11)	−0.0015 (8)	0.0061 (8)	0.0057 (8)
O1B	0.0283 (7)	0.0230 (7)	0.0421 (8)	0.0046 (5)	0.0088 (6)	0.0145 (6)
O2B	0.0258 (8)	0.0334 (8)	0.0699 (11)	0.0072 (6)	0.0095 (7)	0.0238 (8)
O3B	0.0249 (7)	0.0193 (6)	0.0347 (8)	0.0021 (5)	0.0038 (6)	0.0096 (6)
O4B	0.0749 (12)	0.0198 (7)	0.0374 (9)	0.0108 (7)	−0.0020 (8)	−0.0018 (6)
O5B	0.0410 (8)	0.0312 (8)	0.0286 (8)	0.0025 (6)	0.0017 (6)	0.0096 (6)
N1B	0.0316 (9)	0.0214 (8)	0.0275 (9)	0.0006 (7)	0.0003 (7)	0.0060 (7)
C1B	0.0226 (9)	0.0185 (9)	0.0352 (11)	0.0024 (7)	0.0043 (8)	0.0092 (8)
C2B	0.0299 (10)	0.0189 (9)	0.0340 (11)	0.0022 (8)	0.0065 (8)	0.0011 (8)
C3B	0.0288 (10)	0.0239 (9)	0.0266 (10)	0.0000 (8)	0.0056 (8)	0.0041 (8)
C4B	0.0247 (9)	0.0176 (9)	0.0278 (10)	0.0011 (7)	0.0016 (8)	0.0057 (7)
C5B	0.0311 (10)	0.0213 (9)	0.0278 (10)	0.0053 (8)	0.0048 (8)	0.0025 (8)
C6B	0.0319 (11)	0.0274 (10)	0.0281 (10)	0.0041 (8)	0.0081 (8)	0.0065 (8)
C7B	0.0276 (10)	0.0211 (9)	0.0313 (11)	0.0029 (8)	0.0037 (8)	0.0068 (8)
C8B	0.0261 (10)	0.0176 (9)	0.0286 (10)	0.0000 (7)	0.0067 (8)	0.0041 (7)
C9B	0.0285 (11)	0.0262 (10)	0.0382 (12)	0.0015 (8)	0.0016 (9)	0.0020 (9)
C10B	0.0363 (12)	0.0293 (10)	0.0346 (12)	0.0070 (9)	0.0110 (9)	0.0034 (9)
C11B	0.0310 (10)	0.0194 (9)	0.0260 (10)	0.0036 (7)	0.0042 (8)	0.0043 (8)

### Geometric parameters (Å, °)

**Table:**

O1A—C7A	1.366 (2)	O1B—C7B	1.367 (2)
O1A—C1A	1.407 (2)	O1B—C1B	1.406 (2)
O2A—C7A	1.190 (2)	O2B—C7B	1.187 (2)
O3A—C7A	1.316 (2)	O3B—C7B	1.317 (2)
O3A—C8A	1.498 (2)	O3B—C8B	1.502 (2)
O4A—N1A	1.223 (2)	O4B—N1B	1.223 (2)
O5A—N1A	1.230 (2)	O5B—N1B	1.228 (2)
N1A—C4A	1.469 (2)	N1B—C4B	1.467 (2)
C1A—C6A	1.374 (3)	C1B—C2B	1.378 (3)
C1A—C2A	1.377 (3)	C1B—C6B	1.381 (3)
C2A—C3A	1.383 (3)	C2B—C3B	1.387 (2)
C2A—H2AA	0.9500	C2B—H2BA	0.9500
C3A—C4A	1.381 (3)	C3B—C4B	1.378 (3)
C3A—H3AA	0.9500	C3B—H3BA	0.9500
C4A—C5A	1.376 (3)	C4B—C5B	1.379 (3)
C5A—C6A	1.384 (2)	C5B—C6B	1.387 (2)
C5A—H5AA	0.9500	C5B—H5BA	0.9500
C6A—H6AA	0.9500	C6B—H6BA	0.9500
C8A—C10A	1.511 (3)	C8B—C9B	1.516 (3)
C8A—C9A	1.519 (3)	C8B—C10B	1.519 (3)
C8A—C11A	1.525 (3)	C8B—C11B	1.528 (2)
C9A—H9AA	0.9800	C9B—H9BA	0.9800
C9A—H9AB	0.9800	C9B—H9BB	0.9800
C9A—H9AC	0.9800	C9B—H9BC	0.9800
C10A—H10A	0.9800	C10B—H10D	0.9800
C10A—H10B	0.9800	C10B—H10E	0.9800
C10A—H10C	0.9800	C10B—H10F	0.9800
C11A—C11A^i^	1.520 (4)	C11B—C11B^ii^	1.521 (4)
C11A—H11A	0.9900	C11B—H11C	0.9900
C11A—H11B	0.9900	C11B—H11D	0.9900
			
C7A—O1A—C1A	116.31 (14)	C7B—O1B—C1B	117.15 (14)
C7A—O3A—C8A	120.17 (14)	C7B—O3B—C8B	120.21 (14)
O4A—N1A—O5A	123.51 (16)	O4B—N1B—O5B	123.32 (15)
O4A—N1A—C4A	118.20 (16)	O4B—N1B—C4B	118.17 (16)
O5A—N1A—C4A	118.28 (17)	O5B—N1B—C4B	118.51 (15)
C6A—C1A—C2A	121.84 (16)	C2B—C1B—C6B	122.14 (16)
C6A—C1A—O1A	118.27 (17)	C2B—C1B—O1B	117.05 (16)
C2A—C1A—O1A	119.75 (17)	C6B—C1B—O1B	120.58 (17)
C1A—C2A—C3A	119.51 (18)	C1B—C2B—C3B	119.05 (17)
C1A—C2A—H2AA	120.2	C1B—C2B—H2BA	120.5
C3A—C2A—H2AA	120.2	C3B—C2B—H2BA	120.5
C4A—C3A—C2A	118.28 (18)	C4B—C3B—C2B	118.51 (17)
C4A—C3A—H3AA	120.9	C4B—C3B—H3BA	120.7
C2A—C3A—H3AA	120.9	C2B—C3B—H3BA	120.7
C5A—C4A—C3A	122.46 (16)	C3B—C4B—C5B	122.79 (16)
C5A—C4A—N1A	118.76 (16)	C3B—C4B—N1B	118.54 (16)
C3A—C4A—N1A	118.74 (17)	C5B—C4B—N1B	118.65 (16)
C4A—C5A—C6A	118.76 (17)	C4B—C5B—C6B	118.48 (17)
C4A—C5A—H5AA	120.6	C4B—C5B—H5BA	120.8
C6A—C5A—H5AA	120.6	C6B—C5B—H5BA	120.8
C1A—C6A—C5A	119.15 (18)	C1B—C6B—C5B	119.02 (18)
C1A—C6A—H6AA	120.4	C1B—C6B—H6BA	120.5
C5A—C6A—H6AA	120.4	C5B—C6B—H6BA	120.5
O2A—C7A—O3A	129.64 (17)	O2B—C7B—O3B	130.08 (17)
O2A—C7A—O1A	124.14 (16)	O2B—C7B—O1B	124.12 (16)
O3A—C7A—O1A	106.22 (15)	O3B—C7B—O1B	105.79 (15)
O3A—C8A—C10A	109.33 (15)	O3B—C8B—C9B	109.34 (14)
O3A—C8A—C9A	110.37 (15)	O3B—C8B—C10B	110.06 (14)
C10A—C8A—C9A	112.18 (17)	C9B—C8B—C10B	112.45 (16)
O3A—C8A—C11A	101.78 (14)	O3B—C8B—C11B	101.83 (14)
C10A—C8A—C11A	110.03 (16)	C9B—C8B—C11B	113.03 (15)
C9A—C8A—C11A	112.64 (16)	C10B—C8B—C11B	109.62 (15)
C8A—C9A—H9AA	109.5	C8B—C9B—H9BA	109.5
C8A—C9A—H9AB	109.5	C8B—C9B—H9BB	109.5
H9AA—C9A—H9AB	109.5	H9BA—C9B—H9BB	109.5
C8A—C9A—H9AC	109.5	C8B—C9B—H9BC	109.5
H9AA—C9A—H9AC	109.5	H9BA—C9B—H9BC	109.5
H9AB—C9A—H9AC	109.5	H9BB—C9B—H9BC	109.5
C8A—C10A—H10A	109.5	C8B—C10B—H10D	109.5
C8A—C10A—H10B	109.5	C8B—C10B—H10E	109.5
H10A—C10A—H10B	109.5	H10D—C10B—H10E	109.5
C8A—C10A—H10C	109.5	C8B—C10B—H10F	109.5
H10A—C10A—H10C	109.5	H10D—C10B—H10F	109.5
H10B—C10A—H10C	109.5	H10E—C10B—H10F	109.5
C11A^i^—C11A—C8A	114.72 (19)	C11B^ii^—C11B—C8B	114.71 (19)
C11A^i^—C11A—H11A	108.6	C11B^ii^—C11B—H11C	108.6
C8A—C11A—H11A	108.6	C8B—C11B—H11C	108.6
C11A^i^—C11A—H11B	108.6	C11B^ii^—C11B—H11D	108.6
C8A—C11A—H11B	108.6	C8B—C11B—H11D	108.6
H11A—C11A—H11B	107.6	H11C—C11B—H11D	107.6

Symmetry codes: (i) −*x*+1, −*y*+1, −*z*; (ii) −*x*+2, −*y*−1, −*z*+1.

### Hydrogen-bond geometry (Å, °)

**Table:**

*D*—H···*A*	*D*—H	H···*A*	*D*···*A*	*D*—H···*A*
C5A—H5AA···O5B^iii^	0.95	2.59	3.112 (2)	115
C5B—H5BA···O5A^iv^	0.95	2.52	3.205 (2)	129
C11A—H11B···O3A^i^	0.99	2.53	2.888 (2)	101
C11A—H11B···O4B^i^	0.99	2.54	3.502 (2)	165
C11B—H11C···O4B^v^	0.99	2.58	3.546 (2)	164
C11B—H11D···O3B^ii^	0.99	2.49	2.862 (2)	102

Symmetry codes: (iii) −*x*+1, −*y*, −*z*; (iv) −*x*+1, −*y*, −*z*+1; (i) −*x*+1, −*y*+1, −*z*; (v) −*x*+2, −*y*, −*z*+1; (ii) −*x*+2, −*y*−1, −*z*+1.

## References

[bb2] Altomare, A., Cascarano, G., Giacovazzo, C., Guagliardi, A., Burla, M. C., Polidori, G. & Camalli, M. (1994). *J. Appl. Cryst.* **27**, 435.

[bb3] Blessing, R. H. (1995). *Acta Cryst.* A**51**, 33–38. 10.1107/s0108767394005726 7702794

[bb1] Nawazish Ali, S., Begum, S., Winnik, M. A. & Lough, A. J. (2008). *Acta Cryst.* E**64**, o281. 10.1107/S1600536807065993 PMC2915334 21200847

[bb4] Nonius (2002). *COLLECT* Nonius BV, Delft, The Netherlands.

[bb5] Otwinowski, Z. & Minor, W. (1997). *Methods in Enzymology*, Vol. 276, *Macromolecular Crystallography*, Part A edited by C. W. Carter Jr & R. M. Sweet, pp. 307–326. New York: Academic Press.

[bb6] Sheldrick, G. M. (2008). *Acta Cryst.* A**64**, 112–122. 10.1107/S0108767307043930 18156677

